# A Randomized, Placebo-Controlled Trial of the Bivalent Killed, Whole-Cell, Oral Cholera Vaccine in Adults and Children in a Cholera Endemic Area in Kolkata, India

**DOI:** 10.1371/journal.pone.0002323

**Published:** 2008-06-04

**Authors:** Dilip Mahalanabis, Anna Lena Lopez, Dipika Sur, Jacqueline Deen, Byomkesh Manna, Suman Kanungo, Lorenz von Seidlein, Rodney Carbis, Seung Hyun Han, Seong Hye Shin, Stephen Attridge, Raman Rao, Jan Holmgren, John Clemens, Sujit K. Bhattacharya

**Affiliations:** 1 Society for Applied Studies, Kolkata, India; 2 International Vaccine Institute, Seoul, Korea; 3 National Institute of Cholera and Enteric Diseases, Kolkata, India; 4 Shantha Biotechnics, Hyderabad, India; 5 University of Gothenburg, Gothenburg, Sweden; 6 Seoul National University, Seoul, Korea; Dartmouth Medical School, United States of America

## Abstract

**Objectives:**

An effective vaccine against cholera has been used for public health purposes in Vietnam since the 1990s. This vaccine was reformulated to meet WHO requirements. We assessed the safety and immunogenicity of the reformulated bivalent (*Vibrio cholerae* 01 and 0139) killed whole cell oral vaccine in a cholera endemic area in Kolkata, India.

**Design:**

Double-blind, randomized, placebo controlled trial

**Setting:**

The trial was conducted in the clinical trial ward of the Infectious Diseases Hospital in Kolkata, India

**Participants:**

The participants were 101 healthy adults (males and non-pregnant females) aged 18–40 years and 100 healthy children (males and non-pregnant females) aged 1–17 years.

**Interventions:**

Participants were randomized to receive either the bivalent killed whole cell oral cholera vaccine or placebo (killed oral *Escherichia coli* K12)

**Outcome Measures:**

For safety: proportion of subjects with adverse events during the duration of study participation. For immunogenicity: Proportion of subjects who had a ≥4-fold rise in serum vibriocidal antibody titers 14 days after the second dose of vaccine or placebo.

**Results:**

Adverse reactions were observed with similar frequency among vaccine and placebo recipients in both age groups. Among adults 4% of vaccine and 8% of placebo recipients and among children 4% of vaccine and 2% of placebo recipients had at least one adverse event within 28 days of the first dose of the vaccine. Following immunization, 53% of adult and 80% of children vaccinees showed a ≥4 fold rise in serum *V. cholerae* O1 vibriocidal antibody titers. A less pronounced response to *V. cholerae* O139 vibriocidal antibody titers post-immunization was noted among vaccinees.

**Conclusions:**

We found the vaccine to be safe and immunogenic in a cholera-endemic area in India.

**Trial Registration:**

ClinicalTrials.gov NCT00119197

## Introduction

The World Health Organization (WHO) advocates the use of oral cholera vaccines in the control of cholera in addition to other control measures [1]. Only one internationally licensed oral cholera vaccine is available but this remains prohibitively expensive for routine use in cholera-endemic countries. Vietnam produces a two-dose, oral killed whole cell cholera vaccine that has been given through its public health system and is currently produced at about US$0.40 per dose. This bivalent (*Vibrio cholerae* 01 and 0139) vaccine and its monovalent (O1) predecessor have been found to be safe and to confer significant protection against El Tor cholera in both children and adults [2]–[4]. Since 1997, more than 9 million doses of this bivalent vaccine have been administered in Vietnam. No serious adverse events have been associated with this vaccine [5].

In order to expand the use of the oral cholera vaccine globally, the Diseases of the Most Impoverished (DOMI) Programme of the International Vaccine Institute (IVI) decided to support reformulation of the vaccine to comply with WHO standards. The reformulated vaccine was shown to be safe and immunogenic among Vietnamese adults [6]. Prior to technology transfer to a developing country producer outside Vietnam, we assessed the safety and immunogenicity of this reformulated vaccine in a cholera endemic area in Kolkata, India.

## Methods

### Participants

The study was conducted in the clinical trial ward of the Infectious Diseases Hospital in Kolkata, India. The trial protocol was approved by the of the Ethics Committee of the National Institute of Cholera and Enteric Diseases (NICED), the Health Ministry Screening Committee of India and the Institutional Review Board of the IVI in Seoul. The study was monitored by an independent Data Safety and Monitoring Board (DSMB) who reviewed the safety data among adults before proceeding to recruitment of children. We recruited healthy adults (males and non-pregnant females) aged 18–40 years followed by healthy children (males and non-pregnant females) aged 1–17 years. Written informed consent was obtained prior to enrolment and written assent was also obtained from children 12–17 years. Individuals who were pregnant, with abdominal pain, vomiting, loss of appetite, generalized ill-feeling or nausea during the preceding 24 hours; or diarrhea or history of anti-diarrheal or antibiotic use during the past week; or history of diarrhea and abdominal pain lasting for more than 2 weeks during the past 6 months were excluded.

The protocol for this trial and supporting CONSORT checklist are available as supporting information; see Checklist S1 and Protocol S1.

### Interventions

Participants were randomized to receive either 2 doses of the vaccine or placebo, given 14 days apart. Each dose of the vaccine contained 600 ELISA Units (EU) of lipopolysaccharide (LPS) of formalin-killed *V. cholerae* Inaba, El Tor biotype (strain Phil 6973); 300 EU LPS of heat-killed *V. cholerae* Ogawa classical biotype (Cairo 50); 300 EU LPS of formalin killed *V. cholerae* Ogawa classical biotype (Cairo 50); 300 EU LPS of heat-killed *V. cholerae* Inaba, classical biotype (Cairo 48); and 600 EU LPS of formalin-killed *V. cholerae* O139 (4260B). The vaccine had no detectable cholera toxin (1 ng/ml detection limit). The LPS and toxin assays were performed at the University of Gothenburg. All other lot release assays were performed at the Company for Vaccine and Biological Production No. 1 in Hanoi and Shantha Biotechnics in Hyderabad.

The placebo consisted of heat-killed *Escherichia coli* K12 and was identical in appearance to the vaccine. Non-specific LPS content was not measured in the placebo, but this strain has been used in previous oral cholera vaccine clinical trials [3], [6], [7]. Both placebo and vaccine were packaged as liquid formulations in identical vials containing five 1.5-ml doses and were stored at 4–8°C before administration. The vaccine or placebo was given in two doses separated by a two week interval and administered by oral syringe without a needle after which each participant was offered a glass of water. No buffer was co-administered.

### Objectives

We investigated whether the reformulated killed oral cholera vaccine was safe and immunogenic among adults and children residing in a cholera-endemic area.

### Outcomes

The primary endpoints of the study were as follows: for safety, the proportion of subjects with diarrheal adverse events during the study period and for immunogenicity, the proportion of subjects exhibiting 4-fold or greater rises in titers of serum vibriocidal antibodies relative to baseline, 14 days after the second dose of either the killed oral cholera vaccine or placebo. Additional analyses were performed to compare all adverse events during the study period as well as geometric mean serum vibriocidal titers at baseline and 14 days after dose 2 among vaccine and placebo recipients.

Participants were enrolled in the clinical trial ward of the Infectious Diseases Hospital in Kolkata. Adverse events were solicited for 3 days after the first dose. Two weeks after the first dose was given, subjects returned to the clinic for the second dose and were asked to follow-up daily for 3 days. During each follow-up day, study physicians conducted a structured interview regarding the participants' over-all level of activity and bowel movements as well as occurrence of symptoms such as diarrhea, abdominal pain, loss of appetite, nausea, general ill feeling, fever, headache or vomiting during the previous 24 hours. For young children, parents or caretakers were interviewed. Diarrhea was defined as 3 or more loose or liquid stools in a 24 hour period. Two weeks after each dose was given, subjects were asked about any illness that may have occurred, and treatment received, during the interval period. Serious adverse events (SAEs) were defined as any medical events which were incapacitating or preventing normal activities and included death, life-threatening events, hospitalization, and disability.

Blood samples were obtained prior to the first dose and 14 days after the second dose. Sera were separated, shipped frozen and stored at −70°C until paired testing was performed. The microtiter technique was used to detect serum vibriocidal antibodies to *V. cholerae* O1 El Tor Inaba strain (T19479) at the IVI in Seoul as previously described [8]. For the serum vibriocidal antibodies to *V. cholerae* O139, a modified microtiter assay was performed at the University of Gothenburg as previously described [9] using a spontaneously streptomycin-resistant variant derived from the capsule-deficient CIRS134 [[10] (concentration 2×10^5^ ) as indicator strain. Two-fold serial dilutions of pre- and post vaccination sera were performed in duplicates, and the mean of the two determinations was the final titer. The assay was repeated if a ≥2-fold difference was noted between the results of the duplicate tests. Testing was performed by technicians blinded to the study agent received by the subjects. Initial serum dilutions for testing were 1∶2.5 for *V. cholerae* O1 and 1∶10 for *V. cholerae* O139, respectively. Vibriocidal titers <2.5 for *V. cholerae* O1 and <10 for *V. cholerae* O139 were considered as 1.25 and 5, respectively, for statistical analyses. “Seroconversion” was defined as a four-fold or greater increase in titer between pre-vaccination and post-vaccination sera.

### Sample size

The “non-inferiority” approach using a 1 –sided 95% confidence interval was used to calculate the sample size since this allowed us to rule out clinically unacceptable high rates of diarrheal adverse event occurring during the 3 days after either dose as well as establish that the vaccine induced adequate seroconversion to *V. cholerae* O1 Inaba among recipients [11]. Assuming a 10% diarrheal rate among placebo and vaccine recipients alike, to exclude a vaccine-placebo difference in the rate of diarrhea of greater than 20% (upper boundary of the 1-tailed 95% confidence interval) with a power of 0.9, the minimum number of subjects required for each group was 39. For serum vibriocidal responses, assuming a background rate of 5% seroconversion among placebo recipients after the second dose and a true vibriocidal response in the vaccine group of 60%, to exclude a vaccine-placebo difference of 30% (lower boundary of the 1-tailed 95% confidence interval) with a power of 0.9, the minimum of subjects required for each group was 40. To adjust for the number of persons expected to drop out of the study, at least 50 persons were therefore required in each group.

### Randomization –Sequence generation

Separate randomization lists for adults and children were prepared by a statistician in IVI who was otherwise not involved in the study. Randomization numbers were generated in blocks of 8 using the program Visual Fortran 5.0. (Digital, USA) For the children's study, to ensure that each age group was represented, approximately 33–34 children of each age group (1 to 5 years, 6 to 10 years and 11 to 17 years) were randomized to receive either vaccine or placebo.

### Randomization – Allocation Concealment

Study agents were coded using 8 letters (4 for vaccine and 4 for placebo) in the adult trial and 8 different letters in the pediatric trial. Only the code letters on the vials identified the study agents as vaccine or placebo. The codes were revealed to the researchers once recruitment, data collection, and laboratory analyses were complete.

### Randomization – Implementation

The agent administered was determined according to the randomization list. The study nurse or medical officer randomized the participants according to the next available number on entry into the trial. The randomization numbers were linked to one of the 8 letter codes for the adult study and one of the 8 letter codes for the children's study.

### Blinding

The vials were labeled by letter codes at Shantha Biotechnics in Hyderabad according to the list prepared by the statistician at IVI. All study personnel and participants were blinded to treatment assignment during the duration of the study.

### Statistical Methods

Data was entered in Visual Fox Pro ™ v 7.0 (Microsoft Corp, USA) and analyses were performed using Stata™ v 9.0 (Stata Corp., USA). Analyses for contrasts of dichotomous outcomes such as adverse events and seroconversion were performed with the chi-square test, except when data was sparse for which Fisher's exact test was used. For contrasts of vibriocidal titers, Student's t-test was performed. Serum vibriocidal titers and fold rises were logarithmically transformed prior to statistical analyses.

To assess vaccine versus placebo geometric mean fold rises in serum antibody titers, after controlling for potentially confounding variables, multiple linear regression models were fitted. In the models, the logarithm of the serum vibriocidal titer at the post-vaccination bleed was the dependent variable, and vaccination status (vaccine versus placebo), together with the logarithm of the titer of baseline serum vibriocidal antibodies and other potentially confounding variables were fitted as independent variables. In these models we exponentiated the coefficient for the vaccination variable to estimate the ratio of geometric mean-fold rise of serum antibodies in vaccinees to rises in placebo-recipients, after controlling for the potentially confounding variables in the models. Standard errors of the coefficients were used to estimate P values and 95% confidence intervals.

Contrasts of the primary outcomes, diarrhea following dosing and serum vibriocidal seroconversion, were evaluated with 1-tailed P values and 1-tailed 95% confidence intervals. Statistical evaluations of all other comparisons were 2-tailed.

## Results

### Participant Flow and Recruitment

**Figure 1:**
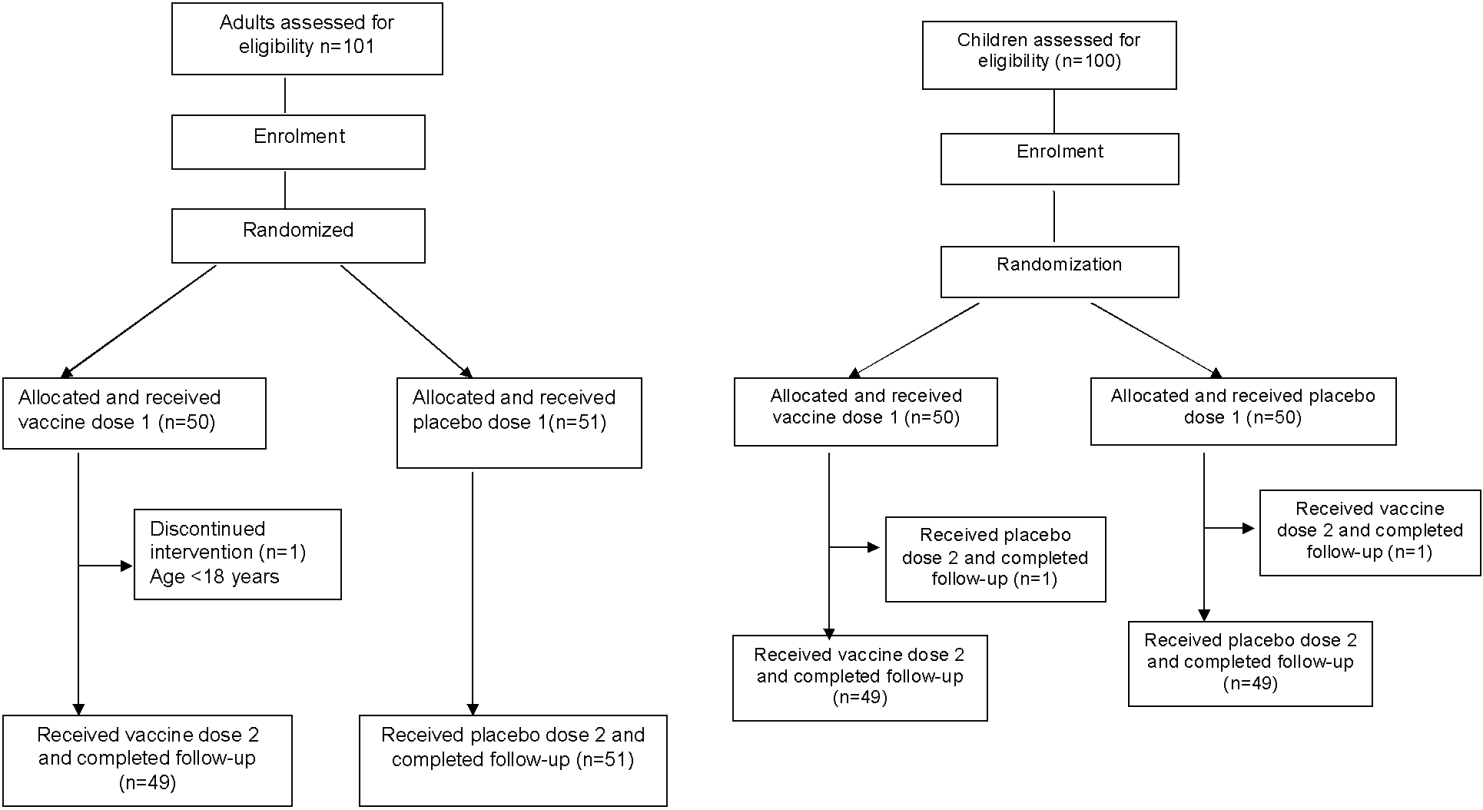
Flowchart of adult and children participants in the study.

The flow of subjects in the adults and children trials is shown in Figure 1. Adult participants were recruited from August to September 2005 and children were recruited from September to October 2005. We enrolled 101 adults who received at least one dose of either vaccine or placebo. On review after the first dose, 1 participant was found to be ineligible because he was 17 years of age at enrollment. Of the 100 eligible adults, all (51 vaccinees and 49 placebo recipients) received the assigned two dose regimen and were followed up with a second blood collection. Subsequently, 100 children were enrolled, of whom 49 received 2 doses of vaccine and 49 received 2 doses of placebo. One child participant randomized to receive vaccine was given placebo for the second dose and one child participant randomized to receive placebo was given vaccine for the second dose. All children were able to completely swallow the study agent dose and were followed up with a second blood collection.

### Baseline Data

Table 1 shows the characteristics of subjects in the vaccine and placebo groups. The mean age of adult vaccinees was 26.8 years (range 18–40) compared to 27.5 years (range 18–40) among placebo recipients. The mean age of children vaccinees was 8 years (range 1–16) compared to 8.5 years (range 1–16) of placebo recipients.

**Table 1:** Characteristics of adult and children vaccine and placebo recipients

	*Adults*		*Children*	
Characteristics	Vaccine	Placebo	Vaccine	Placebo
	n = 51	n = 50	n = 50	n = 50
Age				
Mean (SD)	26.8 (6.9)	27.5 (7.2)	8.0 (4.3)	8.5 (5.1)
Median	25	27	7.5	8
Sex				
Male	25 (49%)	24 (48%)	31 (62%)	21 (42%)

### Numbers Analyzed

For safety, intention-to-vaccinate analysis was performed which included all subjects randomized in the study who received one or more dose of any study agent. 101 adults and 100 children were included in this analysis. For immunogenicity, per-protocol analysis was performed on randomized, eligible subjects who received 2 doses of the correct study agent and were available until the last follow-up. 100 adults were included for analysis of seroconversion to *V. cholerae* O1 and *V. cholerae* O139. For children, 98 were included for analysis of seroconversion to *V. cholerae* O1 but due to small amounts of sera, only 88 sera were available for testing to *V. cholerae* O139.

### Outcomes

No adverse event occurred more frequently in the vaccine than in the placebo group (Table 2). There were 4 serious adverse events (SAE) in 2 vaccinees (1 child, 1 adult) and 2 placebo recipients (both children) who were hospitalized for vomiting and dehydration. All four had eaten a local fermented rice preparation a few hours prior to the onset of vomiting. All other adverse events were mild. The 95% one-sided confidence intervals for the occurrence of diarrheal events excluded more than a 19% greater occurrence among adult vaccinees and more than an 18% greater occurrence among children vaccinees than in placebo recipients in each age group.

**Table 2:** Comparison of solicited adverse events among adults and children following receipt of the first and second doses of vaccine and placebo

	Adults			Children		
	Vaccine N (%)	Placebo N (%)	P Value	Vaccine N (%)	Placebo N (%)	P Value
**Within 3 days after the first dose**	**N = 50**	**N = 51**		**N = 50**	**N = 50**	
Diarrhea	1 [18%] a	0		1 [9%]	0	
Abdominal Pain	1	1		0	0	
Loss of appetite	1	0		0	0	
Nausea	1	1		0	0	
Vomiting	2	1		1	2	
Fever	0	0		1	0	
Headache	0	0		0	0	
General ill feeling	1	0		0	0	
**Within 3 days after the second dose**						
Diarrhea	0	0		0	0	
Abdominal Pain	0	0		0	0	
Loss of appetite	0	0		0	0	
Nausea	0	0		0	0	
Vomiting	0	0		0	0	
Fever	0	2		0	0	
Headache	0	1		0	0	
General ill feeling	0	0		0	0	
**No (%) with** ≥ **one adverse event within 3 days of the first dose**	2 (4) b	2 (4)	1	2 (4)	2 (4)	1
**No (%) with** ≥ **one adverse event within 3 days of the second dose**	0 (0)	2 (4)	.50	0 (0)	0 (0)	1
**No (%) with** ≥ **one adverse event within 28 days of the first dose**	2 (4)	4 (8)	.68	2 (4)	1 (2)	.56
**No (%) with a serious adverse event within 28 days of the first dose**	1 (2)	0 (0)	.50	2 (4)	1 (2)	.56

a Values in brackets represent the upper boundaries for one tailed 95% confidence interval for the differences in the diarrheal adverse events among vaccinees compared to placebo recipients

b Values in parenthesis represent the percentage of group total

There were statistically significant differences in the baseline geometric mean titers (GMT) of vibriocidal antibodies to *V. cholerae* O1 Inaba between adults and children, (P = <0.01) with titers being lower in children (Table 3). The baseline vibriocidal antibody titer to *V. cholerae* O1 Inaba ranged from <2.5 to 5120 in adults and <2.5 to 1280 in children. There were no statistically significant differences in the baseline geometric mean vibriocidal antibody titers to *V. cholerae* O139 between the two age groups, (P = 0.098). Baseline vibriocidal antibodies to *V. cholerae* O139 ranged from 5 to 7650 in adults and 5 to 3200 in children.

**Table 3:** Serum vibriocidal antibody titers to *V. cholerae* O1 Inaba and *V. cholerae* O139 at baseline and 14 days after the second dose among subjects with paired blood specimens

	*V. cholerae O1*			*V. cholerae O139*		
Adults	Vaccine recipients n = 49	Placebo recipients n = 51	P value	Vaccine recipients n = 49	Placebo recipients n = 51	P value
GMT^a^						
Bleed 1	251.6	143.5	0.13	189.4	201.6	0.79
Bleed 2	1127	164.4	<0.01	307.5	234.3	0.16
GMF-rise^b^ (95% CI)	4.5 (3.1, 6.5)	1.1 (1.1, 1.3)	0.04	1.6 (1.4, 2.0)	1.2 (1.0, 1.4)	<0.01
No. of subjects who seroconverted^c^	26 (53%)	0	<0.01	5 (10%)	1 (2%)	0.11
95% CI lower boundary^d^	36%	–				
**Children**	**Vaccine recipients n = 49**	**Placebo recipients n = 49**	**P value**	**Vaccine recipients n = 45**	**Placebo recipients n = 43**	**P value**
GMT^a^						
Bleed 1	32.4	23.7	0.48	116.9	126.6	0.78
Bleed 2	407	25.1	<0.01	291.1	151.7	<0.01
GMF-rise^b^ (95% CI)	12.6 (7.4, 21.3)	1.1 (1.0, 1.2)	<0.01	2.5 (1.9, 3.3)	1.2 (1.1, 1.4)	<0.01
No. of subjects who seroconverted^c^	39 (80%)	1 (2%)	<0.01	12 (27%)	1 (2%)	<0.01
95% CI lower boundary^d^	60%	–				

a Geometric mean reciprocal titer for the cited bleed

b Geometric mean-fold rise between first and second bleed.

c Number of subjects with ≥4 fold rise in titers between first and second bleed

d Lower boundary of the one-tailed, 95% confidence intervals for the percentage of vaccinees that seroconverted compared to the placebo recipients (shown because the primary hypotheses for the trial included this lower boundary, see text).

Table 3 shows that the differences in the geometric mean fold (GMF) rises of *V. cholerae* O1 Inaba vibriocidal antibody titers among vaccinees versus placebo recipients in both adults and children were statistically significant. Multiple linear regression models adjusting for baseline titers revealed similar findings and remained statistically significant (P<.01). Among vaccinees, there was a 4.5 fold rise among adults, and among children there was a 12.3 fold rise. Over-all, there was a 7.4 fold-rise, 14 days after receipt of the second dose.

Seroconversion rates were higher for vaccinees than for placebo recipients in both adults (53% vs. 0%; P<.01) and children (80% vs. 2%; P<.01). The 95% confidence intervals for differences in seroconversion rates between vaccine and placebo recipients excluded a 36% or lower response rate in adults and 60% or lower response rate in children.

Although seroresponses to *V. cholerae* 01 were higher in children than in adults, it was noteworthy that among adult vaccinees with baseline titers ≤80, 11 (85%) seroconverted and a 19- fold rise in serum antibodies from baseline was noted. Similarly, among children vaccinees with such low baseline titers, 29 (85%) seroconverted, and a 23-fold rise in serum antibodies from baseline was observed (see Table 4). Correspondingly, multivariable models demonstrated that differences between vaccinees and placebo recipients in geometric mean-fold rises in serum vibriocidal antibodies and in rates of seroresponses were inversely related to baseline serum vibriocidal titers.

**Table 4:** Frequency table of fold-increase of serum vibriocidal antibody titers to *V. cholerae* O1 Inaba and O139 from baseline and 14 days post-second dose among adult and children vaccinees

	Fold increase in titers from baseline to 14 days after receipt of dose 2 among vaccinees																		
	**Adults**																		
**Baseline Titers**	*V. cholerae* O1											*V. cholerae* O139*							
	≤1	2	4	8	16	32	64	128	256	512		≤1	2	4	8	16	32	64	≥128
≤10					1					1					1	1			
20												1		1					
40			1	1	1	3	2						6	1					
80		2			1							2	3						
160		3	2	3	1							7	8	1					
320	1	6	3	1								10	2						
640	1	1	4									4							
≥1280	4	5	1									1							
	**Children**																		
**Baseline Titers**	***V. cholerae*** ** O1**											***V. cholerae*** ** O139** *							
	≤1	2	4	8	16	32	64	128	256	512	≥1024	≤1	2	4	8	16	32	64	≥128
≤10	4			2	2	1	2	2		1	2				2	2			
20					1	1	2	1				1		2	2	1			
40		1		2	1	1							4	1	1				
80				2	3	1		1				1	5	1					
160		2	2	1	2							4	3						
320			3	1								6	4						
640	1		2									3							
≥1280	2											2							

* Titers to V. cholerae O139 were rounded up

The GMF-rises from baseline of serum vibriocidal antibodies to *V. cholerae* O139 in vaccinees compared to placebo recipients in both adults and children (Table 3) were less pronounced compared to the responses seen to O1 Inaba, although these were statistically significant (P<.01). Multiple linear regression models adjusting for baseline titers revealed that among vaccinees, there was a 1.4 fold rise among adults, a 2 fold rise among children and a 1.6 fold rise overall in vibriocidal titers to O139, 14 days after receipt of the second dose. These findings were statistically significant (P<.01). However, the rates for seroconversion between vaccinees and placebo recipients were only different significantly in children (P<.01).

## Discussion

This is the first study to show that a 2-dose regimen of a recently reformulated Vietnamese oral killed whole cell cholera vaccine is safe, well-tolerated, and immunogenic in a cholera-endemic area. It is interesting to compare serum anti-O1 Inaba vibriocidal antibody responses to this vaccine in this study with those observed in an earlier study of an identical regimen of this vaccine in SonLa, Vietnam, where cholera is not endemic [6]. Overall geometric mean-fold rises in serum antibodies were lower in Kolkata (4.5-fold in adults and 12.6-fold in children) than in SonLa (26.8-fold) where only adults were studied. It was noteworthy, however, that higher baseline antibody titers were noted in Kolkata than in SonLa. Indeed, among subjects in Kolkata with low baseline titers (≤80), a 19-fold rise from baseline was noted in adults and a 23-fold rise from baseline was seen in children. These observations, together with the analyses in the present study showing that responses to the vaccine were inversely related to baseline serum vibriocidal antibody titer, suggest that the higher pre-existing vibriocidal antibody titers in Kolkata than in SonLa explained the lower overall responses seen in the present study [7], [12], [13].

A previous trial found similar serum vibriocidal responses to *V. cholerae* O1 Inaba induced by 2 dose regimens of the internationally licensed, oral B subunit killed whole cell vaccine and the previous version of the Vietnamese oral killed whole cell vaccine [3]. The responses to the reformulated vaccine evaluated in the present study were higher than those seen after either of these two earlier vaccines, both in populations with endemic cholera and without endemic cholera. For example, in Bangladesh the internationally licensed oral B-subunit and whole cells elicited a 4-fold or greater increase in serum vibriocidal antibodies to O1 after the second dose in 43% of adults and in 30% of children, in contrast to the figures of 53% and 80% of the corresponding age groups in the present study done in Kolkata [7]. While definitive conclusions about the comparative immunogenicity of these 3 vaccines would require direct, head-to-head comparisons, it is interesting to note that the reformulated and re-standardized version of the Vietnamese vaccine had an almost 40% increase in the quantities of LPS antigen as compared to the earlier Vietnamese vaccine.

Serum vibriocidal responses to *V. cholerae* O139 were lower in the present study than those observed for the previous formulation of the bivalent Vietnamese vaccine [3], [6]. Although the vaccine vs. placebo differences in the geometric mean fold rise were statistically significant in both adults and children, the rise in antibodies was substantially less compared to *V. cholerae* O1 Inaba antibody titers. The baseline vibriocidal GMT to O139 of both adults and children in our study was >100, higher than those seen in Bangladesh [7], [10] suggesting that our subjects may have been primed by exposure to *V. cholerae* O139 or to other organisms with cross-reacting O antigens. These higher baseline titers may have contributed to the poorer anti-O139 vibriocidal responses in our study. However even for those subjects with lower baseline titers (≤80), only 4 out of 11 adults and 12 out of 23 children seroconverted. This lower vibriocidal response to *V. cholerae* O139 in the present study may have been due to differences in the assays used. In an earlier study of the previous Vietnamese vaccine 2 indicator strains were used in the vibriocidal assays to the O139 serogroup [3]; in the present study we used a different indicator strain. Although serum vibriocidal antibodies to O1 have been regarded as an indirect immunologic correlate of vaccine protection against this serogroup of cholera, the utility of results from vibriocidal assays to O139 is still debated [10], [14]. The presence of a capsular polysaccharide in *V. cholerae* O139 may interfere with the induction or detection of immune response and detection of vibriocidal antibodies [10], [14], [15] . Whether the lower vibriocidal responses to *V. cholerae* O139 in our study indicate that the O139 component of this vaccine elicits poorer immunogenic response, that the differences could have arisen due to differences in the sensitivity of the vibriocidal assay used in our study, remains to be seen and might only be resolved by vaccine efficacy studies. A community-based, randomized, placebo-controlled efficacy trial of a 2-dose regimen of the reformulated Vietnamese oral cholera vaccine is currently underway in approximately 70,000 adults and children in Kolkata. If the vaccine is found safe and protective, this could pave the way for the wide use of this vaccine in the control of cholera worldwide.

## Supporting Information

Protocol S1 Trial Protocol.
(0.24 MB DOC)

Checklist S1 CONSORT Checklist
(0.05 MB DOC)
