# Supplementary material for: A Randomized, Placebo-Controlled Trial of the Bivalent Killed, Whole-Cell, Oral Cholera Vaccine in Adults and Children in a Cholera Endemic Area in Kolkata, India
Source: PLoS One. 2008 Jun 4;3(6):e2323. doi: 10.1371/journal.pone.0002323 (PMC2396289; doi:10.1371/journal.pone.0002323)
Supplement: Protocol S1 — Trial Protocol. (0.24 MB DOC) [file pone.0002323.s001.doc]

**Safety and Immunogenicity Trials of a**

**Killed, Oral Cholera Vaccine in Indian Subjects**

**in Eastern Kolkata, West Bengal**

| **COLLABORATORS:** | Indian Council of Medical Research, Ansari Nagar, New Delhi 110029, Telephone (91-11) 2651-7204, Fax (91-11) 2686-8662 / National Institute of Cholera and Enteric Diseases, Beliaghata, Kolkata 700010, Eastern Kolkata, West Bengal, India, Telephone (91-33) 2350-1176, Fax (91-33) 2353-2524  Shantha Biotechnics PVT.LTD., Serene Chambers, 3rd Floor, Road No. 7, Banjara Hills, Hyderabad 500 034 India, Telephone (91-40) 23543010, Fax (91-40) 23541713  International Vaccine Institute, Seoul National University Campus, Shilim Dong, Kwanka-Ku, Seoul, Korea Telephone (82-2) 872-2801, Fax (82-2) 872-2803 |
| --- | --- |
| **Investigational product:** | Bivalent, Killed, Whole-cell Oral Cholera Vaccine |
| **Control Product:** | Killed oral *Escherichia coli* K12 strain placebo |
| **Principal Investigator:** | Dr Sujit Kumar Bhattacharya, National Institute of Cholera and Enteric Diseases |
| **Co-Investigators :** | Dr Dilip Mahalanabis, Society for Applied Studies  Dr Dipika Sur, Dr Byomkesh Manna, Dr Alok Deb, Dr Swapan Kumar Niyogi, National Institute of Cholera and Enteric Diseases |
| **On-site clinical monitor:** | Dr Asoke Chowdhury |
| **IVI SCIENTISTS:** | Dr John D Clemens, Dr Jacqueline Deen, Dr Rodney Carbis, Dr Lorenz von Seidlein, Dr Carolina Danovaro |
| **Statisticians:** | Dr Byomkesh Manna, Dr Eunsik Park |
| **DATA MANAGEMENT:** | Dr Byomkesh Manna, Ms Sriboni Boral, Dr Eunsik Park, Ms Hyang-sook Park |
| **Trial site:** | National Institute of Cholera and Enteric Diseases, Beliaghata, Kolkata 700010, Eastern Kolkata, West Bengal, India, Telephone (91-33) 2353-7519, Fax (91-33) 2353-2524 |
| **Biological laboratory:** | c/o Prof Jan Holmgren, University of Gothenburg, Sweden |
| **Data Safety Monitoring Board** (**DSMB):** | Dr Bernard Ivanoff, Dr Roger Glass, Dr Jack Lee, Dr Ellwyn Griffiths |
| **Date:** | February 2004 |

**Contact information**

| **Principal and Other Investigators:** | | | |
| --- | --- | --- | --- |
| Dr Sujit Kumar Bhattacharya | National Institute of Cholera and Enteric Diseases | P-33,C.I.T Road, Scheme XM, Beliathata, Kolkata 700 010 | (91-33) 2353-751  [bsujit@vsnl.net](mailto:bsujit@vsnl.net) |
| Dr Dilip Mahalanabis | Society for Applied Studies | 108, Manicktala Main Road, Flat 3/21, Kolkata - 54 | (91-33) 2321-4204 office  (91-33)2337-2562 residence |
| Dr Dipika Sur | National Institute of Cholera and Enteric Diseases | P-33,C.I.T Road, Scheme XM, Beliathata, Kolkata 700 010 | (91-33) 2363-1222  [dipikasur@hotmail.com](mailto:dipikasur@hotmail.com) |
| Dr Byomkesh Manna | - do - | - do - | (91-33) 2363-1222  [mannab2000@yahoo.co.in](mailto:mannab2000@yahoo.co.in) |
| Dr Alok Deb | - do - | - do - | (91-33) 2350-1176  [adeb02@yahoo.com](mailto:Adeb02@yahoo.com) |
| Dr Swapan Kumar Niyogi | - do - | - do - | (91-33) 2350-1176  [niyogisk@hotmail.com](mailto:niyogisk@hotmail.com) |
| **On-site clinical monitor:** | | | |
| Dr Asoke Chowdhury | BA-146, Sector-I, Salt Lake, Kolkata-64, (91-33) 2337-0660 | | |
| **DSMB:** | | | |
| Bernard Ivanoff | Former Medical Officer, Vaccine Research & Development  World Health Organization, Geneva, Switzerland | | |
| Roger Glass | Chief of Viral Gastroenteritis Section  Center for Disease Control & Prevention, USA | | |
| Jack Lee | Chair Professor, Quantitative Medicine, Hanyang University  Westat, Korea | | |
| Ellwyn Griffiths | Former Coordinator, Quality and Safety of Biologicals.  World Health Organization, Geneva, Switzerland | | |
| **Multi-Country Study Coordinators (International Vaccine Institute):** | | | |
| Dr John D Clemens  Dr Jacqueline Deen  Dr Rodney Carbis  Dr Lorenz von Seidlein  Dr Eunsik Park  Dr Carolina Danovaro  Mr Jung-hoon Han  Ms Hyang-sook Park | International Vaccine Institute | Kwanak PO Box 14  Seoul 151-600  Korea  Tel (82-2) 872-2801  Fax (82-2) 872-2803 | [jclemens@ivi.int](mailto:jclemens@ivi.int)  [jdeen@ivi.int](mailto:jdeen@ivi.int)  [rcarbis@ivi.int](mailto:rcarbis@ivi.int)  [lseidlein@ivi.int](mailto:lseidlein@ivi.int)  [espark@ivi.int](mailto:espark@ivi.int)  [cdanovaro@ivi.int](mailto:cdanovaro@ivi.int)  [jhhan@ivi.int](mailto:jhhan@ivi.int)  [hspark@ivi.int](mailto:Kholloday@ivi.int) |
| **External consultant:** | | | |
| Prof Jan Holmgren | University of Gothenburg, Sweden | | |

**Table of Contents**

|  | **Page** |
| --- | --- |
| STUDY SYNOPSIS AND FLOW CHART | 5 |
| ABBREVIATIONS AND DEFINITIONS | 6 |
| 1.INTRODUCTION | 7 |
| 1.1 Background | 7 |
| 1.2 Study Rationale | 8 |
| 2. STUDY OBJECTIVES | 8 |
| 3. STUDY DESIGN AND AGENTS | 8 |
| 3.1 Type of study | 8 |
| 3.2 Eligibility criteria | 9 |
| 3.3 Study vaccine and placebo | 9 |
| 3.4 Packaging and coding | 10 |
| 3.5 Investigational product accountability | 10 |
| 3.6 Storage conditions | 10 |
| 3.7 Random Allocation | 10 |
| 3.8 Administration | 11 |
| 3.9 Blinding and Code Breaking Procedures | 11 |
| 3.10 Duration of the study period for one subject | 11 |
| 4. STUDY PROCEDURES | 11 |
| 4.1 Assessment after vaccination | 11 |
| 4.2 Schedule of observations and visits | 13 |
| 4.3 Description of observations and visits | 13 |
| 4.4 Laboratory procedures | 13 |
| 4.5 Premature termination | 14 |
| 5. Adverse Events | 14 |
| 5.1 Definitions | 14 |
| 5.2 Reporting of Serious Adverse Events | 14 |
| 6. Data Collection and Management Procedures | 14 |
| 7. STATISTICAL CONSIDERATIONS | 15 |
| 7.1 Endpoints | 15 |
| 7.2 Sample Size Calculation | 15 |
| 7.3 Analysis Plans | 16 |
| 8. MONITORING, AUDITING, INSPECTION | 17 |
| 8.1 Responsibilities of the investigator(s) | 17 |
| 8.2 Responsibilities of the IVI coordinators | 17 |
| 8.3 Responsibilities of the DSMB | 17 |
| 9. ETHICAL AND REGULATORY STANDARDS | 17 |
| 9.1 Ethical principles | 17 |
| 9.2 Laws and regulations | 18 |
| 9.3 Informed consent | 18 |
| 9.4 Institutional Review Committee (IRB) | 18 |
| 10. ADMINISTRATIVE ASPECTS | 18 |
| 10.1 Record retention | 18 |
| 10.2 Publications | 18 |
| 11. REFERENCES | 19 |
| APPENDIX 1. INVESTIGATOR’S STATEMENT | 20 |
| APPENDIX 2. INFORMED CONSENT FORMS | 21 |
| APPENDIX 3. CASE REPORT FORMS | 23 |
| APPENDIX 4. BUDGET | 52 |

##### STUDY synopsis and flow chart

| **TITLE** | **Safety and Immunogenicity Trials of a Killed, Oral Cholera Vaccine in Indian Subjects in Eastern Kolkata, West Bengal** |
| --- | --- |
| **Study Type** | Clinical Trial (Phase I Phase II Phase III Phase IV) |
| **STUDY OBJECTIVES** | The objectives are:   - To confirm the safety and immunogenicity of the bivalent killed whole-cell oral vaccine in adult volunteers in Eastern Kolkata, West Bengal, India. - To confirm the safety and immunogenicity of the bivalent killed whole-cell oral vaccine in children volunteers in Eastern Kolkata, West Bengal, India. |
| **LOCATION** | The study will be conducted at the National Institute of Cholera and Enteric Diseases in Eastern Kolkata, West Bengal, India. |
| **STUDY DESIGN** | Randomized, double-blind, placebo-controlled trials in adults and children. |
| **STUDY SUBJECTS** | Healthy non-pregnant adults, aged 18-40 years will be recruited for the adult study. Healthy children aged 1-17 years will be recruited for the children’s study. |
| **SAMPLE SIZE** | 100 adults and 100 children |
| **STUDY VACCINE** | Bivalent, Killed, Whole-cell Oral Cholera Vaccine |
| **EVALUATION CRITERIA:** | Safety   - Proportion of the subjects with AEs   Immunogenicity   - Seroconversion rate (fourfold rise in antibody titer after vaccination) |
| **STUDY PERIOD** | 6 months (June 2004 to December 2004) |
| Flow Chart   | **Day 0** |  | **Days 1 to 3** |  | **Day 14** |  | **Day 15 to 17** |  | **Day 28** | | --- | --- | --- | --- | --- | --- | --- | --- | --- | | Obtain informed consent  Enrol  Ascertain eligibility  Bleed 1  Give dose 1 |  | In-hospital observation |  | Give dose 2 |  | Follow-up at home |  | Visit all two-dose recipients  Bleed 2  Interval medical history | | |

**ABBREVIATIONS AND DEFINITIONS**

##### CRF – Case Report Form

GMP - Good Manufacturing Practices

##### IVI – International Vaccine Institute

ICMR – Indian Council of Medical Research

IDH – Infectious Diseases Hospital

IRB – Institutional Review Board

##### NICED – National Institute of Cholera and Enteric Diseases

NIHE – National Institute of Hygiene and Epidemiology

VABIOTECH - Company for Vaccine and Biological Production No. 1

SAE – Serious Adverse Event

SHANTA – Shantha Biotechnics Pvt Ltd

*V cholerae – Vibrio cholerae*

WHO – World Health Organization

##### INTRODUCTION

##### Background

Cholera remains a serious public health problem worldwide. In 2002, a total of 142,311 cases and 4, 564 deaths were reported to the World Health Organization (WHO) from 52 countries primarily in Africa, Asia and Latin America. The true figures are likely to be much higher due to underreporting. Besides the high mortality and morbidity figures, cholera outbreaks cause economic and social disruption as well.

In India, cholera is a persistent problem particularly in States such as West Bengal, Maharastra, Andra Pradesh, Tamil Nadu, Karnataka, Delhi, and Kerala (1). Several districts in the country have reported explosive outbreaks of cholera (2-4). Since the dramatic appearance of the new serotype O139 Bengal in 1992 in Madras (5), both *V cholerae* O1 and O139 are present in India.

Provision of safe water and food, establishment of adequate sanitation, and implementation of personal and community hygiene constitute the main public health interventions against cholera. These measures cannot be implemented fully in the near future in most cholera-endemic areas. A safe, effective, and affordable vaccine would be a useful tool for cholera prevention and control.

A parenterally administered killed whole cell cholera vaccine was widely available for many years. However, this vaccine offered at best only about 40-50% protection for about 3 to 6 months and produced unpleasant side effects in many vaccinees. In view of these limitations, the vaccine has not been considered satisfactory for general public health use and in 1973 the 26^th^ World Health Assembly abolished the requirement in the International Health Regulations for a certificate of vaccination against cholera (6).

Considerable progress has been made during the last decade in the development of new generation oral vaccines against cholera. These have already been licensed in some countries and are now being considered for wider public health application (7). The World Health Organization’s attitude towards the use of these new generation cholera vaccines is quite different than that towards the old parenteral vaccine. Cholera immunization is now recommended for travellers to high risk areas, refugee camps and for outbreak response. Furthermore, expanded use of cholera vaccines may be recommended for endemic areas, where there is increasing demand from both low- and middle-income populations.

A monovalent (anti-O1) oral killed cholera vaccine was developed by Prof Jan Holmgren in Sweden and is now licensed to a pharmaceutical company in the United Kingdom. The vaccine consists of inactivated whole cells of *V cholerae* supplemented with a purified recombinant–DNA derived B-subunit of the cholera toxin. Large scale field trials of the vaccine in Bangladesh and Peru showed that both the killed whole cell vaccine containing the B-subunit, as well as the killed whole cell preparation alone, conferred significant protection for recipients for up to 3-5 years depending on age. An initial protection of 85-90% was obtained with the killed vaccine containing the B-subunit but this level of protection declined to about 50% after 6 months. The oral vaccine lacking the B-subunit gave a somewhat lower initial level of protection but after 6 months the protection afforded by the two vaccines was similar (8, 9). The vaccine is licensed in several industrialized countries and is used mainly by travellers. Unfortunately, the vaccine is prohibitively expensive for public health use in developing countries.

Starting in the mid-1980s, following technology transfer from Prof Jan Holmgren, Vietnamese scientists at the National Institute of Hygiene and Epidemiology (NIHE) in Hanoi developed and produced an oral, killed cholera vaccine for the country’s public health programs (10). A two-dose regimen of a first generation monovalent (anti-O1) cholera vaccine produced at US$ 0.10 per dose conferred 66% protection against El Tor cholera in fully immunized adults and children (11). However, this was not a randomised double-blind trial. Subsequently, killed 0139 whole cells were added to the Vietnamese vaccine due to the emergence of the new form of epidemic cholera caused by this serogroup. A study found the bivalent vaccine to be safe and immunogenic in adults and children one year and older (12).

A large-scale field trial of the Vietnamese bivalent (O1 and O139) killed, oral cholera vaccine in non-pregnant persons 1 year and older was conducted in Nha Trang, Vietnam (13). Approximately 340,000 persons (20% between 1 to 3 years of age) were randomised to receive a two dose regimen of the vaccine or placebo. No *V cholerae* was isolated during the surveillance thus effectiveness could not be demonstrated. However, surveillance for potential side-effects showed no difference between the vaccine and placebo groups. The data were reviewed by the Data Safety Monitoring Board convened by the WHO and they concluded that the vaccine is safe.

The Vietnamese vaccine has several distinct advantages over the Swedish vaccine. In contrast to the Vietnamese vaccine, the Swedish vaccine does not confer significant protection against the El Tor biotype in younger children, is prohibitively expensive for use in developing countries, requires a buffer during administration, and due to its B-subunit component has strict cold chain requirements.

Since licensure of the oral cholera vaccine in Vietnam, more than 5 million doses have been administered without any report of serious adverse events. The vaccine is produced according to recommended guidelines (6) at the NIHE’s newly privatized arm, the Company for Vaccine and Biological Production No. 1 (VABIOTECH) in Hanoi. VABIOTECH is working towards WHO Good Manufacturing Practices (GMP) certification, which they hope to receive in the next few years. The vaccine production technology has been transferred to pharmaceutical production facilities in Indonesia and China. In Vietnam, the vaccine is delivered by the Expanded Programme of Immunization through mass vaccination of high risk populations.

The International Vaccine Institute (IVI) has negotiated an agreement between VABIOTECH and Shantha Biotechnics PVT.LTD. VABIOTECH will produce bulk oral killed bivalent cholera vaccine under quality control conditions to be supervised by IVI staff. Shanta will purchase bulk vaccine from VABIOTECH, fill and finish the bulk, and obtain regulatory clearance for use of the vaccine in this Phase II trial and a future Phase III trial. Shanta will also produce the placebo agent for use in the trials. In return, Shanta will obtain the technology for future production of the oral killed bivalent cholera vaccine. Training of staff from Shanta will be conducted at VABIOTECH for vaccine production and at the Department of Microbiology and Immunology, University of Gothenburg (Prof Jan Holmgren).

##### Study Rationale

There is an urgent need to conduct a double-blind randomised Phase III trial in a cholera-endemic area to demonstrate efficacy of the bivalent oral killed cholera vaccine. The trial would be pivotal in the internationalization of the vaccine. The trial would pave the way for the introduction of the vaccine into national immunization programmes in India and other cholera-endemic countries. Before this Phase III trial is conducted, a Phase II trial demonstrating the vaccine’s safety and immunogenicity in Indian adults and children is needed.

##### STUDY OBJECTIVES

2.1. To confirm the safety and immunogenicity of the oral killed bivalent cholera vaccine, in adult volunteers in Eastern Kolkata, West Bengal, India.

2.2. To confirm the safety and immunogenicity of the oral killed bivalent cholera vaccine, in children volunteers in Eastern Kolkata, West Bengal, India.

##### STUDY DESIGN AND AGENTS

##### Type of study

The trials in adults and children will proceed separately but simultaneously. These trials will be randomized, double-blind, and placebo-controlled.

Healthy, non-pregnant adults (aged 18 to 40 years) and children (aged 1-17 years) will be randomized to receive a two-dose regimen of either the bivalent oral killed cholera vaccine or a killed oral *Escherichia coli* K12 strain placebo. Each agent will be administered to volunteers on days 0 and 14; volunteers will be followed-up for 3 consecutive days after each dose; and bled on days 0 and 28. The primary endpoints to be measured are the occurrence of adverse events and serum vibriocidal response.

##### Eligibility criteria

Healthy, non-pregnant adults (aged 18 to 40 years) and children (aged 1-17 years) will be recruited from Kolkata for the trials. The process of obtaining consent, assessing eligibility, administering the vaccine and placebo, and bleeding will occur in sequential steps during one session.

Prior to the screening evaluation, volunteers or their parents or guardians will be:

- informed of the nature of the vaccine and placebo and will be given pertinent information as to the intended purpose of the study, possible benefits and possible adverse experiences.
- informed of the procedures and possible hazards to which he/she or his/her child will be exposed.
- requested to read the informed consent form (see Appendix 2, pages 21-22) or this will be read to him/her.
- requested to affix his/her signature or left thumb print on the form. The consent form will also be signed by a witness and the investigator.
- provided with a copy of the signed informed consent.

Next, all consenting subjects will be assigned a unique Study ID number, in consecutive sequence. This means that all consenting subjects will receive an ID number, regardless of whether they are medically eligible or ineligible. Then all consenting subjects will be examined for study eligibility. Subjects will be excluded if they answer yes to any the following questions:

- During the past week, has the subject had diarrhoea (3 or more loose/watery stools within a 24 hour period)?
- During the past week, has the subject taken any antibiotics?
- During the past week, has the subject taken any anti-diarrhoeal medicines?
- During the past 6 months, has the subject had one or more episodes of diarrhoea lasting for more than 2 weeks?
- During the past 6 months, has the subject had one or more episodes of abdominal pain lasting for more than 2 weeks?
- During the past 24 hours, has the subject had any abdominal pain or cramps, loss of appetite, nausea, general ill feeling, fever or vomiting?
- For adult females, has she missed her menstrual period or is she currently pregnant?

A study physician must review this information and declare the subject eligible or ineligible. An enrolment and eligibility form (see CRF Visit 1, page 25 to 28) will be filled out for each consenting subject, regardless of whether they are ultimately judged eligible for the study.

##### Study vaccine and placebo

Eligible, consenting volunteers will be allocated to one of two groups, receiving one of the following:

*a. Bivalent oral killed cholera vaccine*: each dose of this vaccine contains:

- Inactivated V.Cholerae Inaba (569B), Classical biotype - 25.10^9^ cells
- Inactivated V.Cholerae Ogawa (Cairo 50) Classical biotype - 25.10^9^ cells
- Inactivated V.Cholerae Inaba (Phil 6973) El Tor biotype - 50.10^9^ cells
- Inactivated V.Cholerae O139 - 50.10^9^ cells

A two-dose regimen of a first generation monovalent (anti-O1) cholera vaccine produced at US$ 0.10 per dose conferred 66% protection against El Tor cholera in fully immunized adults and children (11). However, this was not a randomised double-blind trial. Subsequently, killed 0139 whole cells were added to the Vietnamese vaccine due to the emergence of the new form of epidemic cholera caused by this serogroup. A study found the bivalent vaccine to be safe and immunogenic in adults and children (12). A large Phase III trial of the bivalent vaccine was conducted in Nha Trang (Vietnam) starting in 1997. Again, safety and immunogenicity was demonstrated but during the period of follow-up, no cholera cases were detected, so that efficacy could not be shown.

*b. Escherichia coli K12 strain placebo*: each dose of placebo contains heat-killed *E. coli* K12 strain in an amount whose optical turbidity is identical to that for the cholera vaccine.

Each dose of vaccine or placebo is 1.5 ml. The vaccine and placebo will be dispensed in liquid form in identical vials. The merthiolate (as preservative) content will be 0.01% as final concentration. Prior to the trials, aliquots from the individual lots of the cholera vaccine will have undergone extensive quality control testing at VABIOTECH, the University of Gothenburg, and Shanta for sterility, detoxifying agent, thiomersal assay, immunogenicity in animals, functional residual cholera toxin activity (rabbit skin test) and LPS content by an ELISA method using polyclonal antibody. Shanta will issue the final single certificate of analysis for vaccine and placebo.

##### Packaging and coding

The volume of liquid in each five-dose, 10-ml vial would be 8.6 ml, i.e. the five doses (1.5 ml /dose x 5 = 7.5 ml) plus 15% wastage.

For the adult study, each vaccine vial will be coded with one of two letters (e.g. L, O) and the placebo will be coded with one of two different letters (e.g. B, W). Each adult subject will receive an agent coded with the same single letter for the first and second dose (i.e. L, O, B, or W). For the childrens’ study, the vaccine will be coded with one of two different letters (e.g. A, J) and the placebo will be coded with one of two different letters (e.g. S, U). Each child will receive an agent coded with the same single letter for the first and second dose (i.e. A, J, S or U).

Letters with similar appearance (e.g. O and Q) will not be chosen as codes for the vaccine and placebo. Selection of letters and coding of the vaccine and placebo will be done by staff of Shanta headquarters in Hyderabad. The staff at Shanta who will be responsible for affixing letters on each vial of the agents will not be involved in any other way in the conduct of the trial nor will be present at the study site.

##### Investigational product accountability

Shanta will obtain the necessary clearances from the Drug Controller General of India for the import and use in clinical trials of the cholera vaccine. The agents will be kept in a secure place. The investigator or the person in-charge of the product management will maintain records of the product delivery to the trial site, the inventory at the site, the dose(s) given to each subject, and the return of unused doses to Shanta.

##### Storage conditions

The vaccine and placebo will be stored at 4 to 8°C before administration. Vials taken to the field will be carried in vaccine boxes designed for the Expanded Programme of Immunization (EPI). Each box will have a single ice pack, and care will be taken not to freeze the vaccine. Each box will be equipped with a thermal monitor, whose measurement will be recorded upon return from the field. Vials from each lot of vaccine or placebo will be saved for future testing if necessary.

##### Random Allocation

A randomization list will be prepared by IVI. The randomization code will be sequential numbers unique to each individual. Randomization numbers will be generated in blocks 4, which includes two vaccines and two placebos. Age-stratified randomization of the children’s study will be conducted using the following age groups: 1 to 5 years, 6 to 10 years, and 11 to 17 years. The randomization numbers will be linked to one of the 4 letter codes for the adult study and one of the 4 letter codes for the childrens’ study.

##### Administration

After acquisition of informed consent and ascertainment of eligibility, consenting, eligible subjects will be entered into the trial in the randomization sequence, as noted above.

During administration of the agents, 4 vials (one of each of the 4 codes) will be at hand. The agent to be received will be determined according to the randomization list. After vigorous shaking of the vial, 1.5 ml will be extracted into the syringe and gently squirted into the mouth of the recipient, followed by water *ad libitum*. The agent will be administered with a disposable syringe (without needle). If it is judged that a dose is not successfully ingested (e.g., regurgitated or spat out), recipients will be offered a single replacement dose, using the same procedure. Each 5-dose vial will be opened and used up according to the randomization list.

At the time of the first dose, information about vaccine administration will be entered into CRF Visit 1. This information will note the code of the assigned agent, the success of administration as well as certain additional information (see page 25 to 28).

14 days after the first dose, a second dose of the same code will be administered according to the same procedures. The only contraindications to the second dose will be:

- 1. the occurrence, after the first dose, of a severe allergic reaction (generalized urticaria, wheezing, anaphylaxis);
  2. the development of an inter-current illness after the first dose, judged by the Principal Investigator to be too severe to continue participation; or
  3. a diagnosis of pregnancy after the first dose.

At the time of the second dose, a second vaccine administration form (see CRF Visit 5, page 35 to 37) will be completed. If the subject does not take the second dose, this will be noted in the CRF Endpoint (see page 44-46).

Syringes used for vaccination with the vaccine and placebo will be disposed of after each dose, to prevent inadvertent administration of contaminating amounts of non-assigned agents.

##### Blinding and Code Breaking Procedures

Only the code letters on the vials will identify the agents as vaccine or placebo. Since both vaccine and placebo will be identical in appearance and the codes will only be known to the IVI staff labelling the agents, the NICED staff administering the vaccine during the trial and the subjects will be blinded as to the identity of the agents.

The principal investigator at NICED and the IVI will keep the sealed randomization numbers with vaccine groups specified, in case of serious adverse events. The codes can be broken during the study only if the choice of treatment of a serious adverse event is dependent on the subject’s vaccine assignment.

##### Duration of the study period for one subject

The duration of the study period for one subject would be 28 days.

##### STUDY PROCEDURES

##### Assessment after vaccination

a. Follow-up for Adverse Events

After each dose, subjects will be held for observation in the clinic (vaccinating area) for 30 minutes. Any adverse events noted will be recorded on CRF Visit 1 (see page 25 to 28) for dose, and on CRF Visit 5 (see page 35 to 37) for dose 2.

After the first dose, each subject will be observed in hospital for 3 days. CRFs Visits 2 to 4 (page 29 to 34) will be completed to record 24-hour recall histories of symptoms, as well as to measure axillary body temperature. After the second dose, each subject will be followed-up at home for 3 days. CRFs Visits 6 to 8 (page 38 to 43) will be completed to record 24-hour recall histories of symptoms, as well as to measure axillary body temperature. Thus, complete 3-day follow-up after the two doses will entail completion of all six CRFs.

A final visit to each subject will be made at 28 days. Information will be taken about any medical illnesses that may have occurred during the two weeks after the second dose, and this will be entered into CRF Endpoint (see page 44 to 46). Any medications taken will be recorded in CRF Medications, Adverse Events, and Serious Adverse Events (see page 47 to 51).

b. Follow-up for Serious Adverse Events

In addition to active follow-up for adverse events noted within three days of vaccination as described above, any other adverse or serious adverse event which occurs from the beginning of the study up to the end will be reported using CRF Medications, Adverse Events, and Serious Adverse Events (see page 47 to 51).

A serious adverse event (experience) is any untoward medical occurrence that at any dose:

- results in death,
- is life-threatening. The term “life-threatening” in the definition of “serious” refers to an event in which the subject was at risk of death at the time of the event; it does not refer to an event, which hypothetically might have cause death, if it were more severe.
- requires in subject hospitalization or prolongation of existing hospitalization,
- results in persistent or significant disability/incapacity, or
- is a congenital anomaly/birth defect.

Medical and scientific judgment should be exercised in deciding whether expedited reporting is appropriate in other situations, such as important medical events that may not be immediately life-threatening or result in death or hospitalization but may jeopardize the subject or may require intervention to prevent one of the other outcomes listed in the definition above. These should also usually be considered serious.

c. Serology

To ascertain whether serum vibriocidal responses to the vaccine are adequate, blood specimens (7ml for persons > 5 years; 2 ml for younger persons by venipuncture) will be obtained at the time of the first dose and again 14 days after the second dose (e.g., on day 28). Each specimen will be labelled with the following information: date of blood draw, name, and study ID number.

After the first bleed, CRF Visit 1 (see page 25 to 28) will be completed to indicate the success of the blood collection. At the time of second bleed, CRF Visit 5 (see page 35 to 37) will be completed to indicate the success of the blood collection. During the first and second bleeds, numbered laboratory stickers will be affixed to link each form to each specimen.

Blood will be kept refrigerated immediately after collection, and serum will be separated within 4 hours of collection. All serum specimens will be divided into two aliquots, each labelled by date of blood draw, name, study ID number, and aliquot number (1 or 2), and all aliquots will be frozen at -20°C for storage prior to testing.

##### Schedule of observations and visits

| **Day 0**  **(Visit 1)** |  | **Days 1 to 3**  **(Visit 2 to 4)** |  | **Day 14**  **(Visit 5)** |  | **Day 15 to 17**  **(Visit 6 to 8)** |  | **Day 28**  **(Visit 9)** |
| --- | --- | --- | --- | --- | --- | --- | --- | --- |
| Obtain informed consent  Enrol  Ascertain eligibility  Bleed 1  Give dose 1 |  | In-hospital observation |  | Give dose 2 |  | Follow-up at home |  | Visit all two-dose recipients  Bleed 2  Interval medical history |
| Form 1 |  | Forms 4 a to c |  | Form 2 |  | Forms 4 d to f |  | Form 5 |

##### Description of observations and visits

1. Day 0 – Subjects will be enrolled at a special clinic created by NICED at the adjacent Infectious Diseases Hospital (IDH), of which Dr Mahalanabis will be over-all supervisor. Informed consent will be obtained, the coded agent administered, CRF Visit 1 (see page 25 to 28), and blood collected in this clinic.
2. Day 1 to 3 – Subjects will to be admitted to hospital for observation CRFs Visits 2 to 4 (page 29 to 34) completed.
3. Day 14 – Subjects will be requested to return after 2 weeks and will receive the second dose at the clinic. CRF Visit 5 (see page 35 to 37) will be completed.
4. Day 15 to 17 – Subjects will be followed-up at home and CRFs Visits 6 to 8 (page 38 to 43) will be completed.
5. Day 28 - Subjects will be requested to return after 2 weeks for collection of blood. CRF Visit 9 (see page 44 to 46) will be completed.

Follow-up at home will be done for those who do not return to the clinic for the second dose or for collection of blood at day 28.

##### Laboratory procedures

a. Serologic testing – Serum will be tested for vibriocidal antibodies in the laboratory of Professor Jan Holmgren at the University of Gothenburg. All sera will be analyzed in a paired fashion, in order of Study ID number, and all tests will be conducted without knowledge of the agent received by the subject.

Serum vibriocidal antibodies to *V. cholerae* O1 (El Tor Inaba; strain T19479) will be evaluated by a microtitre assay (14). In order to measure vibriocidal antibodies to *V cholerae* O139, O139 vibrios of the partly encapsulated vaccine strain 4260B and the capsule deficient mutant strain M010-T4 provided by the Virus Research Institute, Cambridge, Masachusetts, will be used in a final concentration of 2.5 x 10^5^ bacteria ml ^-1^. Fresh guinea big complement will be added in a fourfold less diluted concentration (1:7.5) than in the O1 vibriocidal assay. The vibriocidal titre will be defined as the highest dilution causing complete inhibition of bacterial growth. Two-fold serial dilutions of prevaccination and postvaccination specimens will be tested side-by-side in duplicates. The titres will be adjusted in relation to a reference serum specimen included in each test to compensate for variations between analyses on different occasions. The antibody titre ascribed to each sample will be the mean of the duplicated determinations, which are not allowed to vary by more than one twofold dilution for either the reference or the test sera. When larger variations occur, the tests will be repeated.

A four-fold or greater increase in titre between prevaccination and postvaccination sera will be taken as indicating seroconversion.

##### Premature termination

Each subject or subject’s parent/guardian is free to accept or reject the proposal to enrol himself/herself/his or her child in this study. Even after enrolment the subject or subject’s parent/guardian will be able to withdraw himself/herself/his or her child from the study at any time.

##### Adverse Events

##### Definitions

An adverse event following immunization is a medical incident that takes place after immunization and causes concern. Adverse events may either be causally or coincidentally related to the vaccination. Possible adverse events would include abdominal pain and cramps, loss of appetite, nausea, general ill feeling, fever and vomiting. Follow-up for adverse events following immunization will be conducted and recorded as described in 4.1a.

# Serious adverse events are those which are incapacitating, preventing normal activities, including death, life-threatening events, hospitalization, disability, and occurrence of malignancy. The relationship of administration of the study agent to the serious adverse event will be assessed as follows:

Not related: The serious adverse event has no relation to administration of the study agent, (i.e. existence of a clear alternative explanation, an unreasonable temporal relationship between the study agent and the event, or non-plausibility).

Unlikely related: A clinical (including a laboratory test) abnormality, with a temporal relationship to administration of the study agent but with an improbable causal relationship, and for which other drugs, chemicals, or underlying disease could provide explanations.

Possibly related: A clinical (including a laboratory test) abnormality, with a reasonable time sequence in relation to administration of the study agent, but which could also be explained by concurrent disease or other drugs or chemicals.

Probably related: A clinical event (including a test abnormality) with a reasonable time sequence in relation to administration of the study vaccine, unlikely to be attributed to concurrent disease or other drugs or chemicals.

Definitely related: A clinical event (including a test abnormality) with a clear time sequence in relation to administration of the study vaccine and likely to be attributed to the study agent.

Unknown: A clinical event (including a test abnormality) which cannot be evaluated because information is insufficient or contradictory, and which can neither be supplemented nor verified.

##### Reporting of Serious Adverse Events

All SAEs will be reported immediately to the local monitor, who will hold the codes to the agents. If deemed necessary by one or more SAEs, the monitor will have the authority to call a temporary moratorium on vaccination, so that DSMB can review the side-effect data. The DSMB will also review the results of the adult study before the trial in children is started.

##### Data Collection and Management Procedures

The Clinical report forms will be completed as detailed above. In addition, the principal investigator will complete a summary form (see Form 6, page 43) for each subject. Study personnel will extract all data collected in Forms 1 to 6 for computerization. Data will be double-entered into computers in a dedicated area located at NICED, using data entry programs specially created for the project by the IVI.

These programs will utilize FOXPRO for Windows; all programs will incorporate range and consistency checks *pari passu* with data entry. This software will provide error reports, exception lists, and summary reports for each activity. The software will also automatically back-up data at systematic intervals onto hard disks and diskettes, and will provide for an audit trail of all sequential changes made.

Data security for this data management system will be augmented by automatic computer virus scanning at start-up of each data entry and data management session, and password protection for accessing data and data management software. In addition, backup files generated by the data management software will be kept in a secure cabinet.

Following entry of the data in Eastern Kolkata, West Bengal, India, the data will be transmitted electronically to the IVI for final edit checks, queries, and archiving.

All data management will be undertaken without access to the vaccine code of study subjects.

##### STATISTICAL CONSIDERATIONS

##### Endpoints

In this trial in adults and in children, we will compare the cholera vaccine and the placebo recipients for:

1) the occurrence of symptoms to show safety; and

2) serum vibriocidal responses (>4-fold and geometric mean-fold) to serogroups O1 and O139 to show immunogenicity.

##### Sample Size Calculation

The sample size for this study is calculated with the assumptions that it is important to evaluate whether: 1) the vaccine induces acceptable serum vibriocidal responses in relation to the placebo group; 2) the vaccine induces acceptable levels of diarrhoeal adverse events in relation to the placebo group. Following a recently published study of killed oral cholera vaccines in Vietnam (13), we make the following assumptions.

# Adult study

For diarrhoeal adverse events in the study, we assume: 1) the background rate of diarrhoea in the placebo group will be 10% after each dose; 2) the true rate of diarrhoea is the same in the vaccine and placebo groups; 3) we wish to exclude, for the vaccine, a one-tailed 95% CI for the vaccine-placebo difference in the rate of diarrhoea of greater than 20%, with .9 power. With these assumptions, and using the method of Blackwelder for precision-based sample size calculations (14), a total of 39 subjects per group would be needed.

For serum vibriocidal responses (defined as >4-fold increases between baseline and post-second dose in either Inaba or Ogawa antibodies) in the study, we assume 1) the background rate of responses in the placebo group will be 5% after the second dose; 2) the true rate of vibriocidal responses in the vaccine groups is 60%; 3) we wish to exclude, for the vaccine, a one-tailed lower 95% CI for the vaccine-placebo difference of lower than 30% with .9 power. With these assumptions, and using the method of Blackwelder for precision-based sample size calculations (14), a total of 40 subjects per group would be needed.

If we assume further that the dropout rate between the first dose and the second bleed will be 20-30%, 50 subjects per group, for a total of 100 entered subjects, will be required.

# Children’s study

For diarrhoeal adverse events in the study, we assume: 1) the background rate of diarrhoea in the placebo group will be 10% after each dose; 2) the true rate of diarrhoea is the same in the vaccine and placebo groups; 3) we wish to exclude, for the vaccine, a one-tailed 95% CI for the vaccine-placebo difference in the rate of diarrhoea of greater than 20%, with .9 power. With these assumptions, and using the method of Blackwelder for precision-based sample size calculations (14), a total of 39 subjects per group would be needed.

For serum vibriocidal responses (defined as >4-fold increases between baseline and post-second dose in either Inaba or Ogawa antibodies) in the study, we assume 1) the background rate of responses in the placebo group will be 5% after the second dose; 2) the true rate of vibriocidal responses in the vaccine groups is 60%; 3) we wish to exclude, for the vaccine, a one-tailed lower 95% CI for the vaccine-placebo difference of lower than 30% with .9 power. With these assumptions, and using the method of Blackwelder for precision-based sample size calculations (14), a total of 40 subjects per group would be needed.

If we assume further that the dropout rate between the first dose and the second bleed will be 20-30%, 50 subjects per group, for a total of 100 entered subjects, will be required.

The required sample sizes are summarized as follows:

|  | **Children** | **Adults** |
| --- | --- | --- |
| **Vaccine group** | 50 | 50 |
| **Placebo group** | 50 | 50 |

##### Analysis Plans

Both intention-to-treat and per-protocol analysis results will be described in the report.

*Intention-to-Treat Analysis (ITT)*

Every subject randomized in the study (who receive the correct or incorrect study agent, one or more doses, and complete or incomplete doses) will be analyzed, except if he/she did not receive any dose of the study agent (vaccine or placebo) or if no post randomization data was collected for this subject.

*Per-Protocol Analysis*

A per protocol analysis will compare subjects according to the study agent actually received and will include only those subjects who satisfied the inclusion/exclusion criteria, followed the protocol, and received two complete, correct doses. The following non-compliant subjects will be excluded:

- Subjects included without meeting at least one inclusion criterion
- Subjects included despite meeting at least one exclusion criterion
- Subjects found non compliant with the blood sampling schedule.
- Subjects vaccinated with the wrong study agent (non compliance with the randomization code).
- Subjects excluded from the ITT analysis.

*Analysis of demographics*

Demographic characteristics of subjects enrolled will be tabulated by group and overall.

*Analysis of safety*

The number and percentage of subjects (with 95% CI) with at least one adverse event (solicited and/or unsolicited) after vaccination and during the 4 weeks follow up period will be compared between the study groups.

The number and percentage of subjects with at least one Serious Adverse Event, with the frequencies of each type of event will be compared between the study groups.

*Analysis of immunogenicity*

Demonstration of at least a fourfold rise in serum anti-O1 and anti-O139 vibriocidal antibody titer will be the measure of vaccine immunogenicity. The number and percentage of adults and children (with 95% CI) who exhibit at least a fourfold rise in serum anti-O1and anti-O139 vibriocidal titer after vaccination will be compared between the study groups.

*Interim analysis*

No interim analysis is planned.

##### MONITORING, AUDITING, INSPECTION

##### Responsibilities of the principal investigator

The principal investigator will conduct the study in accordance with this protocol and will attempt to recruit the required number of patients in a reasonable period of time so as to complete the trial at the earliest. He will provide copies of the protocol to all the members of his study team. He will discuss this material with them and conduct training to assure that all the members of his study team are fully informed regarding the vaccine/placebo and the conduct of the study. He will ensure that all his associates, colleagues and employees assisting in the conduct of this study are informed about their obligations in meeting their respective commitments. He will provide a final report of the study.

The principal investigator will ensure that all case report forms will be completed and computerised in real time (that is within 24 to 48 hours of completion of the form) to assure accurate and timely data. Any forms with queries or inconsistencies noted during data entry will be sent back to the field for correction or clarification.

##### Responsibilities of the IVI coordinators

The coordinators from IVI will ensure that the trial is adequately monitored. At regular intervals, contact with the study site will be made through visits, e-mail, and telephone calls to review the study progress, adherence to the protocol, and any problems. During the monitoring visits, the following will be examined: subject informed consent, subject recruitment and follow-up, vaccine allocation, vaccine storage and transport, follow-up of subjects, and laboratory procedures. The IVI coordinators will discuss any problems with the investigators and define, after deliberation, any action(s) to be taken.

##### Responsibilities of the DSMB

Representatives of the DSMB will visit the study site for quality assurance. They will be independent of and separate from the activities of the IVI staff. They will evaluate trial conduct and compliance with the protocol, the standard operating procedures, Good Clinical Practice, and the applicable regulatory requirements. This may occur at any time from start to after conclusion of the study.

##### ETHICAL AND REGULATORY STANDARDS

##### Ethical principles

The justification for using a placebo in this study is that: 1) no cholera vaccine is currently recommended for use in public health programs in India and 2) a valid assessment of vaccine safety and immunogenicity in India, which is endemic for diarrhoea and for cholera, can only be obtained with use of a placebo. The placebo will consist of heat-killed bacteria (*E. coli* ), which belongs to the normal human gastro-intestinal flora and produces no adverse effects.

Risk-benefit. The cholera vaccine has not been reported to be associated with adverse events in the course of studies involving more than 150,000 vaccinees. The potential benefits to participants are substantial, since cholera is endemic in Eastern Kolkata, West Bengal, India and a vaccine-preventive effect would translate into a real reduction of risk.

Benefit to all participants. To ensure potential benefit to the placebo group, a full course of the vaccine will be offered free of charge to placebo recipients at the conclusion of the study, assuming, as expected, that the vaccine is found to be without significant adverse events. No pro-rated payment will be given, but compensation for transportation and time lost from work will be given. Vitamins or food will be offered after each blood sample is taken.

##### Laws and regulations

The study will be performed in accordance with the Declaration of Helsinki.

##### Informed consent

Informed consent will be obtained from the participants (or the parents/guardians) using the forms shown in Appendix 2 (pages 21 - 22). This agreed upon English version will be translated into Bengali prior to use in the trial.

##### Institutional Review Board (IRB)

Before initiation of the study, the final protocol and the informed consent will require clearance by the Scientific Advisory Committee of the NICED, the Ethics Committee of the Indian Council for Medical Research (ICMR), and the Institutional Review Board (IRB) of the IVI.

##### ADMINISTRATIVE ASPECTS

##### Record retention

The principal investigator and NICED will keep all trial documents for at least 2 years after the completion the study. It is IVI’s responsibility to inform the principal investigator/NICED as to when these documents no longer need to be retained.

##### Publications

NICED, IVI, and Shanta shall jointly own the rights to the data, clinical, and biological specimens, results and other findings resulting from this trial. Parties are encouraged to publish the results of their work in a collaborative fashion for the benefit of the public while taking care to protect the intellectual property rights to proprietary discoveries. There shall be joint access of data. Guidelines for authorship of major, international, peer-reviewed journals will be used to establish authorship. Each party shall provide the others with a copy of each manuscript and abstract at least 30 days before submission for publication in a journal or presentation at an international meeting. The parties will have the right to examine the publication before it is printed and disseminated, and to request changes to the use of their name.

Subject to agreement on a case-by-case basis, each party is encouraged to produce and disseminate electronic versions of important publications produced as a result of this Cooperative Agreement. Each party will permit the others to disseminate such electronic versions as long as all original formatting, credits, and contents are maintained.

The contribution of all parties involved in this Cooperative Agreement shall be acknowledged in all abstracts, reports, or other peer-reviewed scientific publications containing data or information collected during the Project duration.

##### REFERENCES

1. Government of India. Health Information of India 1995, DGHS, New Delhi.
2. Sur D, Dutta P, Nair GB, Bhattacharya SK. Severe cholera outbreak following floods in a northern district of West Bengal. *Indian J Med Res* 2000; 112: 178-82.
3. Radhakutty G, Sircar BK, Mondal SK, Mukhopadhyay AK, Mitra RK, Basu A, Ichpugani I, Nair GB, Bhattacharya SK. Investigation of an outbreak of cholera in Allepey and Palghat districts, South India. *Indian J Med Res* 1997; 106: 455-7.
4. Niyogi SK, Mondal S, Sarkar BL, Garg S, Banerjee D, Dey GN. Outbreak of cholera due to *V cholerae* O1 in Orissa state. *Indian J Med Res* 1994; 100: 217-8.
5. Ramarmurthy P, Garg S, Sharma R, Bhattacharya SK, Nair GB, Shimada T,, Karasawa T, Kurazono H, Pal A, Takeda Y. Emergence of a novel strain of *Vibrio cholerae* with epidemic potential in southern and eastern India. *The Lancet* 1993; 341: 703-4.
6. World Health Organization Expert Committee on Biological Standardization. Guidelines for the production and control of inactivated oral cholera vaccines. 26 – 30 November 2001, Geneva. BS/01/1938.
7. Cholera Vaccines, *Weekly Epidemiological Record*, 2001, 76, 117 – 124
8. Clemens J D et al , Field trials of cholera vaccines in Bangladesh: results from a three year follow- up. *The Lancet* 1990; 335: 270 –3.
9. Sanchez J L, Vasquez B, Beque R et al, Protective efficacy of the oral whole cell / recombinant B subunit cholera vaccine in Peruvian military recruits. *The Lancet* 1994; 344: 1273 – 6.
10. Trach DD. The problem of cholera vaccination in Vietnam. *In:* Prospects for public health benefits in developing countries from new vaccines against enteric infections. Stockholm: SAREC Documentation, 1990.
11. [Trach DD, Clemens JD, Ke NT, Thuy HT, Son ND, Canh DG, Hang PV, Rao MR.](http://www.ncbi.nlm.nih.gov:80/entrez/query.fcgi?cmd=Retrieve&db=PubMed&list_uids=9014909&dopt=Abstract) Field trial of a locally produced, killed,oral cholera vaccine in Vietnam. *The* *Lancet* 1997; 349, 231-5.
12. [Trach DD, Cam PD, Ke NT, Rao MR, Dinh D, Hang PV, Hung NV, Canh DG, Thiem VD, Naficy A, Ivanoff B, Svennerholm AM, Holmgren J, Clemens JD.](http://www.ncbi.nlm.nih.gov:80/entrez/query.fcgi?cmd=Retrieve&db=PubMed&list_uids=11884967&dopt=Abstract) Investigations into the safety and immunogenicity of a killed oral cholera vaccine developed in Viet Nam. *Bulletin of the World Health Organization* 2002; 8, 2-8.
13. Clemens JD, Holmgren J, Trach DD. Evaluation of the bivalent, killed oral cholera vaccine produced in Vietnam and tested in a large-scale field trial in Nha Trang, Vietnam. In preparation.
14. Jertborn M, Svennerholm A-M, Holmgren J. Saliva, breast milk, and serum antibody responses as indirect measures of intestinal immunity after oral vaccination or natural disease. *Journal of Clinical Microbiology* 1986; 24: 203-9.
15. Blackwelder WC. Showing a treatment is good because it is not bad: when does "noninferiority" imply effectiveness? *Control Clin Trials*. 2002 Feb;23(1):52-4.

##### 
